# Systematic Review of the Application of Computational Fluid Dynamics for Adult Aortic Diseases

**DOI:** 10.31083/j.rcm2412355

**Published:** 2023-12-19

**Authors:** Jian Song, Shiqi Gao, Enzehua Xie, Wei Wang, Lu Dai, Rui Zhao, Chenyu Zhou, Juntao Qiu, Cuntao Yu

**Affiliations:** ^1^Department of Vascular Surgery, Fuwai Hospital, National Center for Cardiovascular Diseases, Chinese Academy of Medical Sciences and Peking Union Medical College, 100037 Beijing, China; ^2^Department of Cardiac Surgery, Fuwai Hospital, National Center for Cardiovascular Diseases, Chinese Academy of Medical Sciences and Peking Union Medical College, 100037 Beijing, China; ^3^National Clinical Research Center for Cardiovascular Diseases, Fuwai Hospital, National Center for Cardiovascular Diseases, Chinese Academy of Medical Sciences and Peking Union Medical College, 100037 Beijing, China

**Keywords:** computational fluid dynamics, aorta aneurysm, aorta dissection, biomechanics

## Abstract

**Background:**

Computational fluid dynamics (CFD) is a new medical method combining medicine and science. The aim of this study is to summarize and analyze the application of CFD in adult aortic diseases.

**Methods:**

This systematic review followed the Preferred Reporting Items for Systematic Reviews and Meta-Analyses (PRISMA) guidelines. A search in the PubMed, Cochrane Library and Chinese databases identified 47 highly relevant articles. Studies were included if they assessed biomechanical markers and their potential association with progression or rupture of aortic aneurysms or dissections.

**Results:**

There are no randomized controlled trials to examine the direct relationship between all biomechanical parameters and aortic disease progression or rupture. Wall stress and peak wall rupture risk can predict the risk of aortic aneurysm rupture using biomechanics, which is more accurate than the prediction based on “diameter” alone. Areas with lower time averaged wall shear stress (TAWSS) and higher oscillatory shear index (OSI) are at risk for further aortic expansion or dissection. Higher relative residence time (RRT) area can predict platelet activation and thrombosis. In addition, pressure, flow field and other indicators can also roughly predict the risk of aortic disease progression.

**Conclusions:**

Contemporary evidence suggests that CFD can provide additional hemodynamic parameters, which have the potential to predict the progression of aortic lesions, the effect of surgical intervention, and prognosis.

## 1. Introduction

Aortic diseases include aortic dissection (75%), intramural hematoma, penetrating aortic ulcer, aortic aneurysm, coarctation of the aorta, and congenital aortic arch dysplasia [1]. The first three of the above diseases are referred to as acute aortic syndromes. These syndromes are characterized by acute onset, high mortality, and poor prognosis [2]. Recently, it has been determined by cardiac surgeons that preventing and treating aortic diseases requires systematic research on their occurrence, risk assessment, and treatment methods, entailing the combination of epidemiology, biology, computational mathematics, computational simulations, and other technologies.

Computational fluid dynamics (CFD) has emerged as an important tool in the development of new energy sources, the manufacturing of large-scale equipment, and research in aerospace navigation. CFD is a branch of fluid mechanics integrated with mathematics and computer science. It obtains its corresponding mechanical index parameters by solving equations when the fluid flows in a specific area and certain boundary conditions are met. Currently, with the development of Digital Imaging and Communications in Medicine (DICOM), CFD has become a powerful tool for diagnosing and treating aortic diseases such as aortic dissections [3, 4], thoracoabdominal aneurysms [5, 6], artificial blood vessel evaluation [7, 8], outlining plans for surgery [9], and determining surgical outcomes [8, 10, 11]. CFD utilizes data from computed tomography angiography (CTA) and magnetic resonance imaging (MRI) to obtain a 3-dimensional (3D) reconstruction of aortic vessels, which entails numerical simulation, solution of Navier-Stokes equations, and subsequent visualization. From this reconstruction, multiple hydrodynamic indices are able to be obtained, allowing for the analysis of microscopic fields for blood flow and determining the blood flow status for branching vessels, as well as examining liquid-structure interface interactions and outlining patient prognoses for normal, sub-healthy, diseased or postoperative aortas.

## 2. Methods

This systematic review was conducted in accordance with the Preferred Reporting Items for Systematic Reviews and Meta-Analyses (PRISMA) guidelines. Our literature search used “computational fluid dynamics”, “aortic disease”, “aortic dissection”, “aortic aneurysm”, and “hemodynamics” as keywords and “(computational fluid dynamics) AND (aortic disease)” as the basic retrieval formula. We found numerous publications in PubMed as well as the Chinese (National Knowledge Infrastructure, CNKI and Wanfang Data) and Cochrane Library databases, with a total of 405 articles in English and 54 in Chinese.

Based on these publications, we applied the following inclusion and exclusion criteria. The inclusion criteria, in which CFD was used, were: (1) Hydrodynamic changes in adult healthy/diseased aorta. (2) Biomechanical risk factors for aortic dissection, as well as aortic aneurysm progression or rupture. (3) Effectiveness of artificial blood vessels and surgical methods. (4) Adverse biomechanical factors affecting long-term outcomes of aortic diseases, and (5) Differences in CFD analyses, based on MRI, CTA, transthoracic echocardiogram (TTE), or other methods. Additionally, a high level of medical evidence, as well as citation indices for the published journals, was taken into account as part of the inclusion criteria. The exclusion criteria were as follows: (1) Studies involving children with congenital aortic dysplasia. (2) Effectiveness on internal and external tunnel reconstruction by CFD, for simulating congenital heart disease surgery. (3) Aortic/cardiac pathologies caused by valvular disease. (4) Research direction focused on improving numerical simulation algorithms, or the proposal and establishment of new simulation models, and (5) Poor research quality, such as only simply describing CFD and hemodynamics, as well as lacking meaningful conclusions or predictive findings.

**Fig. 1.**
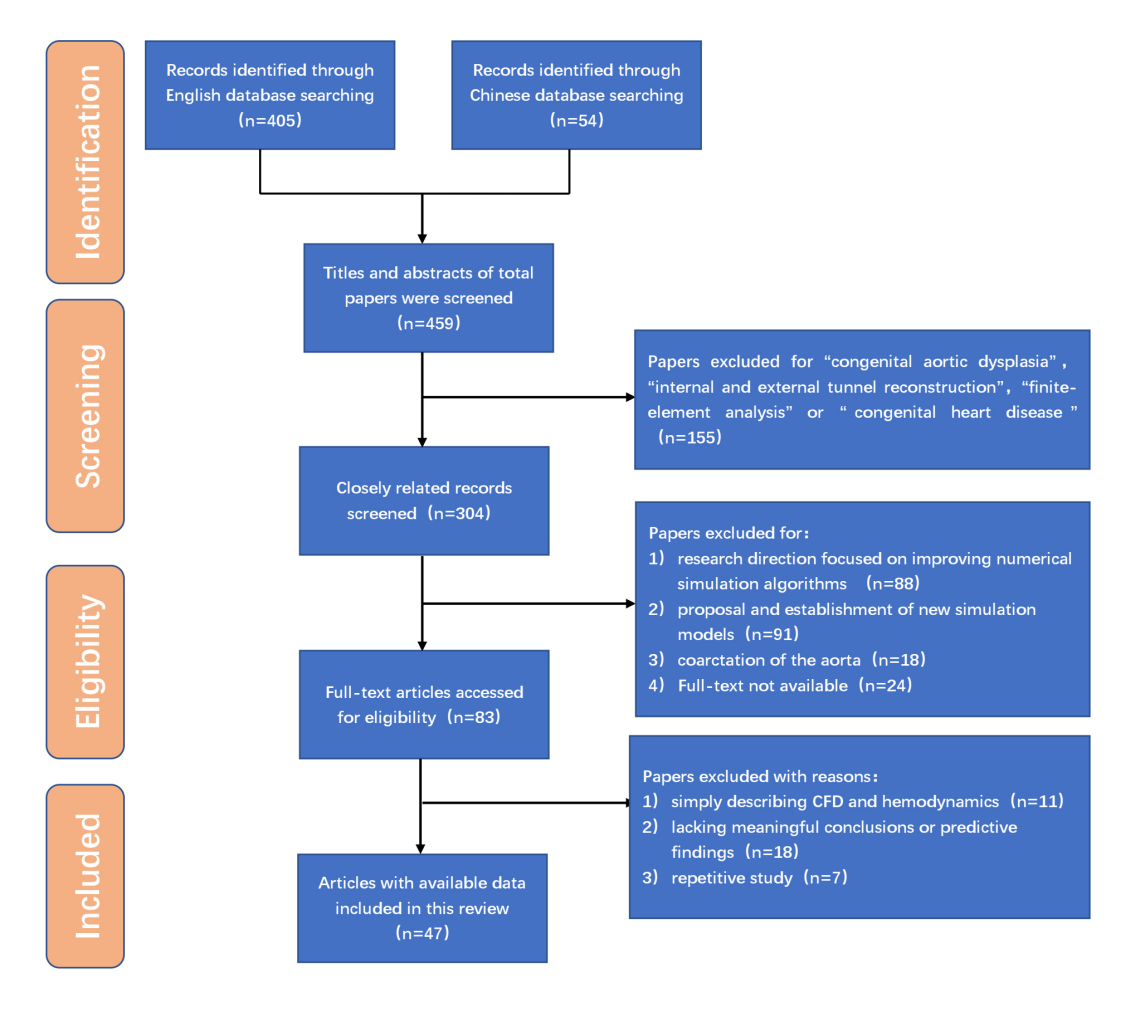
**Flow chart of search strategy**.

After applying both inclusion and exclusion criteria, 3 Chinese- and 48 English-language publications, from January 1997 to February 2022, were included in this review. A literature search, as well as application of inclusion and exclusion criteria, were conducted independently by 2 researchers, and disagreements were resolved by a separate third investigator (Fig. 1).

## 3. Results

### 3.1 The Development of Computational Fluid Dynamics in its Application for Aortic Diseases

The dynamics of aortic blood flow have been examined as far back as the 16th century, when Leonardo da Vinci postulated that, based on its morphology, the sinus of Valsalva, a region of the aortic root, may play a crucial role in initiating retrograde blood flow and specific vortices after aortic valve closure [12]. Centuries later, in 1856, Rudolf Virchow, the German “father of pathology”, also noticed a spatial relationship between abnormal blood flow and atherosclerosis [13]. Subsequently, in the 20th century, multiple biomechanical researchers, radiologists, and surgeons conducted multi-dimensional studies on aortic blood flow and the fluid-structure interface of mechanical parameters. For instance, Friedman* et al*. [13] created a silicone model of an aorta, obtained from an autopsy of a 63-year-old male with moderate atherosclerosis, and used laser Doppler to measure fluid velocity and wall shear force. They found that different wall shear levels were associated with intimal thickening [13]. Another study by Chang* et al*. [14] used MRI to describe the flow field within both true and false lumens (FLs) of aortic dissections. Vorp* et al*. [15] used computer simulation technology to reconstruct two 3D models, and demonstrated that both shape and diameter were essential for predicting the progression of abdominal aortic aneurysms.

The application of CFD technology to the aorta was first facilitated by the data collected by Long* et al*. [16, 17, 18, 19], who successively conducted hemodynamic studies on superior arch branches, the descending aorta, and key abdominal aortic branches. Their data served as the basis for boundary setting, which was then utilized by subsequent numerical simulation studies based on CTA [16, 17, 18, 19]. Animal experiments, using pigs, were first conducted in 2000 by Angouras* et al*. [20], where they found that hypertensive states increased aortic medial stiffness and generated wall shear stress (WSS), eventually leading to aortic dissection. In 2009, Doyle* et al*. [21] developed a silicone blood vessel to simulate the elastic parameters of the human aorta, in order to define the structural properties of the wall of an aortic aneurysm. They noted that the use of wall stress was more able to accurately predict the risk of rupture for abdominal aortic aneurysms [21]. Karmonik* et al*. [4, 22, 23, 24, 25] conducted a series of studies on Stanford type B aortic dissections, which demonstrated the feasibility of using CFD for analyzing aortic dissection and the hydrodynamic factors contributing to type B dissection events. In addition, intraoperative simulation of the extent of tear coverage and thoracic endovascular aortic repair (TEVAR), as well as prognosis, were also studied, thereby serving as the foundation for applying CFD in decision-making under surgical simulation training [4, 22, 23, 24, 25].

### 3.2 Approaches for Implementing Computational Fluid Dynamics

**Fig. 2.**
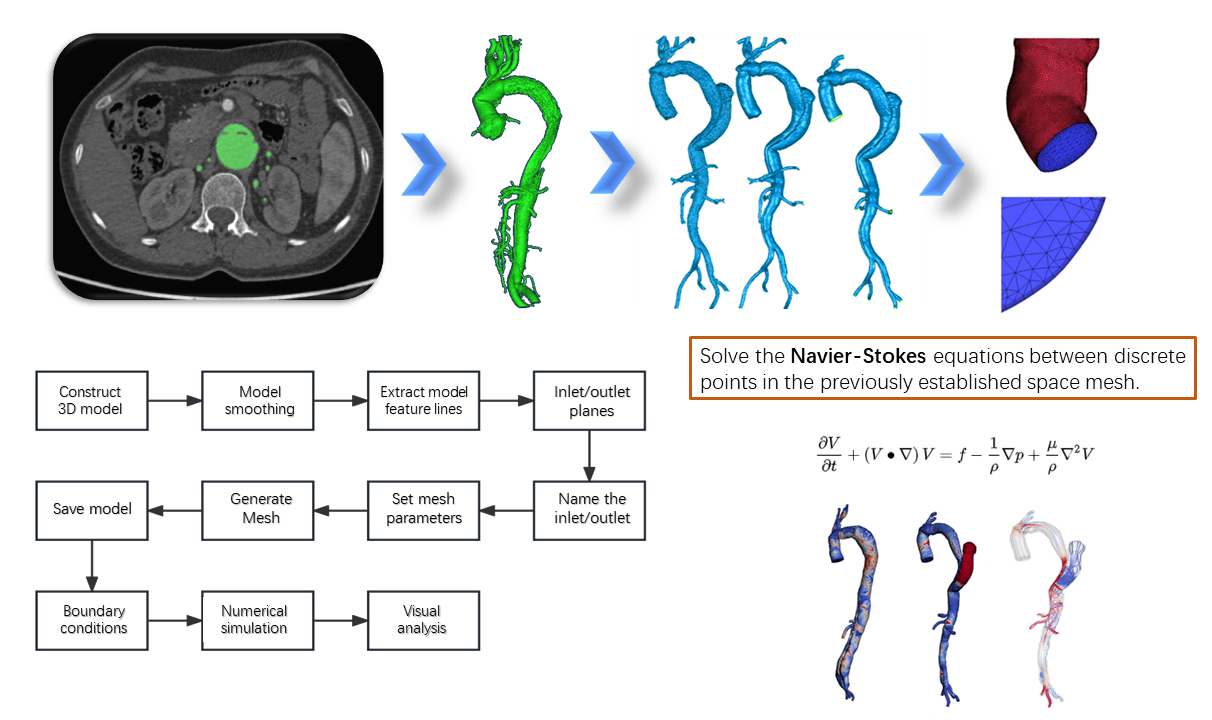
**Diagram of the steps for performing a CFD analysis**. CFD, computational fluid dynamics; 3D, 3-dimension.

There are several major steps involved in implementing CFD. The brief process is shown in Fig. 2.

#### 3.2.1 Obtain Medical Image Data and Build 3D Models 

The actual morphology of the aorta is first obtained by CTA or MRI. The flow velocity is also obtained by PC-MRI, which can be used to specify boundary conditions later. We usually get DICOM format. Medical image processing software such as Mimics Research 19.0 (Materialise’s Interactive Medical Image Control System, Materialise Group, Leuven, Belgium) is then used to generate 3D geometric models of the aorta combined with semi-automatic segmentation, region growth tool and manual processing.

#### 3.2.2 Model Smoothing and Mesh Generation 

Engineering software such as Geomatic studio (Geomagic Group, San Francisco, CA, America), ANSYS Workbench (Design modeler, ANSYS Group, Canonsburg, PA, America) is used to smooth the edges of the model to make it more consistent with the real surface state of blood vessels. In addition, blood flow inlets and outlets need to be specified for the model to make it a tubular device that can be used for analysis. Due to the complex topology of the aorta, ICEM CFD software (ANSYS Group, Canonsburg, PA, America) is generally used for volume grid calculation. Tetrahedral or polyhedral mesh are generated inside the blood vessels, and triangular or prismatic surfaces are formed at the boundaries. The number of mesh should ensure that the general index difference does not exceed 5%.

#### 3.2.3 Setting Boundary Condition 

The vessel wall of the aortic model is generally considered to be non-slip rigid, and the blood is assumed to be an incompressible Newtonian fluid with a density of 1044 kg/$m^{3}$ and a dynamic viscosity of 0.00365 kg/m/s. This is the general boundary condition of most researches, and specific researches may change according to the actual situation. The flow at each inlet and outlet can be directly imported according to MRI, measured according to Doppler ultrasound, or obtained according to previous studies.

#### 3.2.4 Numerical Simulation and Visual Analysis 

Numerical simulation is to establish the variable relationship between discrete points in the previously established space mesh and solve the algebraic equation between them. Specific methods can be used include finite element method (FEM), finite difference method (FDM), boundary element method (BEM), finite volume method (FVM) and finite analytic method (FAM). At present, it can be automatically solved and post-processed by commercial software such as ANSYS Workbench (Design simulator, ANSYS Group, Canonsburg, PA, America). And can be solved by software such as Ansys CFD (ANSYS Group, Canonsburg, PA, America) post in the form of visual expression of mechanical parameters.

### 3.3 Application of Computational Fluid Dynamics for Identifying Parameters Predictive of Aortic Aneurysm Progression and Rupture

Currently, evaluating the risk of aortic rupture is mainly based on a single morphological index, namely aortic diameter; in patients without high-risk factors, such as Marfan’s syndrome or other familial genetic disorders, having a maximum diameter of ≥5.5 cm is an indication for surgical intervention. However, even for patients with a diameter of <5.5 cm, the probability of adverse events, such as rupture, is still 5–10% [26]. With an increased understanding of unilateral morphological indicators, it is inadequate to only use a diameter < or ≥5.5 cm to predict the risk of aortic rupture and the necessity for surgical intervention. A combination of biomechanical factors is considered the most reliable method for determining whether an individual is at risk for developing an aortic rupture [27]. The significance of different biomechanical factors is shown in Table 1 (Ref. [5, 6, 28, 29, 30, 31, 32, 33, 34, 35, 36, 37, 38]).

**Table 1.** **Characteristics of individual studies associated with aortic aneurysm**.

Authors	Year of publication	No. of cases	Imaging data	Modelling and simulation methods	CFD parameter	Key findings
Bluestein* et al*. [32]	1997	An *in vitro* flow pattern	DPI	FEM	Activation parameter	Actual deposition onto the wall was dependent on the wall shear stress distribution along the stenosis, increasing in areas of flow recirculation and reattachment.
Jesty* et al*. [33]	2003	An *in vitro* test	Flow cytometry	CFD	Shear stress	Exposure of platelets to shear conditions on the same order as found in the vasculature causes significant platelet activation, and that this activation is dependent on both shear stress and time of exposure.
Les* et al*. [29]	2010	8	MRI	FEM	WSS	Exercise may positively alter the hemodynamic conditions hypothesized to induce aneurysm growth. The low, OSI, flow seen at rest, which is hypothesized to be associated with aneurysm growth, was largely eliminated during exercise.
					OSI	
					TKE	
Suh* et al*. [34]	2011	8	MRI	FEM	PRT	A long-duration PRT region localized in the aneurysm which may represent flow stagnation and recirculation zone with elevated probability of platelet aggregation and adhesion.
Hardman* et al*. [6]	2013	3	CTA	LES	NWPRT, TAWSS	Peak monocyte residence
			PC-MRI	DPM		time increases with aneurysm size, and mean residence
						time increases rapidly above a sac diameter of 1.8 times the inlet diameter, which suggests there may be a critical aneurysm size above which monocyte infiltration, and therefore wall degradation, increases significantly.
Jayendiran* et al*. [35]	2020	4	MRI	FVM	WSS	The change in aortic geometry of ATAA subjects showed decreased WSS, TAWSS, elevated OSI, RRT, viscosity, and RPI near the ascending aortic region compared to healthy subjects.
					TAWSS	
					RRT	
					RPI	
Joly* et al*. [5]	2020	41	CTA	FVM	OSI, WSS, RRT, ECAP	The risk prediction model based on hemodynamics is better than that based on morphological indicators alone.
Meyrignac* et al*. [37]	2020	81	CTA	FEM	WSS	Combined analysis of lumen volume and wall shear stress was associated with enlargement of abdominal aortic aneurysms at 1 year, particularly in aneurysms smaller than 50 mm in diameter.
Zhou* et al*. [38]	2020	38	CTA	FEM	WSS	Aortic aneurysm rupture did not occur in the high shear stress area, but in the low shear stress area.
					Thrombus index	
Bappoo* et al*. [31]	2021	295	CTA	CFD	TAWSS	Aneurysms within the lowest tertile of shear stress, versus those with higher shear stress, were more likely to rupture or reach thresholds for elective repair.
					OSI	
					RRT	
Etli* et al*. [28]	2021	3	CTA	FEM	WSS	It was found that abnormal changes in WSS and higher pressure load may lead to rupture and risk of further dilatation.
			ECHO		Area-weighted average wall Y+	
Salmasi* et al*. [36]	2021	10	MRI	CFD	WSS	Elevated WSS also predicted a reduction in elastin levels and lower SMC count. And there is an association between elevated WSS values and aortic wall degradation in ATAA disease.
					TAWSS	
Petuchova* et al*. [30]	2022	2	CTA	FEM	WSS	The aneurysm-based model demonstrates a 45% greater wall displacement, while the oscillatory shear index decreased by 30% compared to healthy aortic results.
				CMM-FSI	OSI	

CTA, computed tomography angiography; FVM, finite volume method; OSI, oscillatory shear index; WSS, wall shear stress; ECAP, endothelial cell activation potential; LES, large eddy simulation; DPM, discrete phase modelling; NWPRT, near-wall particle residence time; FEM, finite element method; ECHO, echocardiography; MRI, magnetic resonance imaging; TKE, turbulent kinetic energy; CMM-FSI, fluid-structure interaction with coupled momentum method; DPI, digital particle image; RPI, wall rupture index; CFD, computational fluid dynamics; PRT, particle residence time; PC-MRI, phase-contrast magnetic resonance imaging; TAWSS, time-averaged wall shear stress; RRT, relative residence time; SMC, smooth muscle cell; ATAA, ascending thoracic aortic aneurysms.

A prevailing biomechanical factor is WSS, which was found in one study to be significantly lower in dilated ascending aortic aneurysms, compared to aortas from normal individuals without aneurysms. This, coupled with peak systolic pressure load, was 18.56–23.8% higher in the dilated segment of the aorta, indicating that the combination of lower WSS and higher pressure could contribute to increased risk for further expansion and aortic aneurysm rupture [28]. This association between lower WSS and higher pressure with aortic rupture has been supported by multiple other studies, in which lower WSS or time averaged WSS (TWSS), with higher oscillatory shear index (OSI), were more prone to expansion, dissection or rupture [29, 30, 39, 40], compared to normal areas. However, most of these studies were limited by being comparative in nature, and only focusing on fluid dynamics indicators for specific patient groups, leading to selection biases. A comprehensive study was performed in 2021 by Bappoo* et al*. [31], which included 295 patients with an abdominal aortic aneurysm, to which CFD was applied. More importantly, a longer median follow-up period of 914 days was conducted, and additional clinical baseline data, such as age, gender, baseline diameter, blood pressure, and smoking history, were included in the prediction model. In this study, lower WSS was identified as an independent risk factor for abdominal aortic aneurysm progression, and adverse events were more prevalent (44%), compared to those with intermediate (27%) and high WSS (29%; all *p* = 0.010) [31]. WSS anomalies, along with higher relative residence time (RRT), was also associated with platelet aggregation and activation, which promotes thrombus formation in aneurysms [32, 33, 34]. Abnormal WSS can also affect the arrangement and morphology of endothelial cells and stimulate cytokine secretion, which can increase intercellular permeability, promote inflammatory cell adhesion and local oxidative stress, rendering the region more prone to future focal dissections [35]. In contrast, a higher WSS was associated with reduced elastin and smooth muscle cells, leading to stiffer, less compliant aortic walls, eventually resulting in thinning, dilation, and rupture [6, 36]. Based on these findings, a predictive model combining clinical baseline characteristics, along with morphological and mechanical indicators, would significantly improve the identification of individuals with a high risk for aortic aneurysm progression and rupture, especially among those with maximum aortic diameters <5 cm [5, 37].

### 3.4 Application of Computational Fluid Dynamics for Evaluating Type B Aortic Dissection

According to the International Registry of Aortic Dissection (IRAD), the incidence of type B aortic dissection was about 33%, of which 57–63% were the uncomplicated type. The in-hospital mortality rate was approximately 10% [41]; however, it is approximately 25% among discharged patients who have undergone conservative treatment, with an approximately 66% chance of aortic-related adverse events [42]. As a result, CFD could serve as a useful tool for developing timely surgical interventional strategies, via its analyses of true/false luminal hemodynamic performance, as well as aiding in the early detection of adverse events, such as significant progression, poor organ perfusion, reverse tear to type A dissection, and threatened rupture (Table 2, Ref. [4, 9, 22, 43, 44, 45, 46, 47, 48, 49, 50, 51]). Karmonik* et al*. [4] conducted a series of studies on type B dissection, using CFD technology. They found, based on reconstructions and simulations of the blood flow field using MRI, that the false luminal blood flow was disordered and turbulent, particularly at the aortic location proximal to the primary rupture. Furthermore, false luminal pressure was ten times higher than the true lumen, serving as the basis for the false lumen being more at risk for further expansion. WSS was also lower in the false lumen, which favors thrombosis formation [4]. A follow-up study had shown that higher WSS was present around the primary tear, and further expansion of the false lumen resulted in decreased flow velocity and pressure, along with increased turbulence, which may serve as a self-compensatory mechanism, but at the expense of an increased risk of rupture [43]. A significant increase in pressure difference, in terms of ascending versus descending aortas, and true versus false lumens, among type B dissection patients, compared to healthy controls, was also observed in a comparative study. The study also postulated that this significant difference could serve as a marker of abnormal abdominal organ perfusion [22], which is further supported by a research group from China. This team demonstrating that significant pressure differences between true and false lumen was indicative of poor prognoses [9, 52]. These findings, however, were contradicted by the findings from Long* et al*. [50], who conducted CFD with finite element analysis based on CTA on 3 patients, and found that the progression of descending aortic aneurysms was not associated with pressure difference changes, but with larger WSS differences between the true and false lumen. This is due to the pressure differences between true and false lumen in the distal descending aorta being close to 0 or a negative value. Furthermore, the flaps in this region are generally thicker and less mobile, making it more difficult for the true lumen to be constricted.

**Table 2.** **Characteristics of individual studies associated with TBAD**.

Authors	Year of publication	No. of cases	Imaging data	Modelling and simulation methods	CFD parameter	Key findings
Karmonik* et al*. [4]	2009	1	MRI	CFD	WSS	Complex flow patterns in the false lumen - as visualized by the blood flow vectors in combination with low velocity magnitudes indicating almost stagnant flow — may be able to predict thrombus formation, even more so if WSS magnitude is low on the aortic wall.
					TAWSS	
					OSI	
Rudenick* et al*. [46]	2010	3	Ideal *in vitro* model	FEM	WSS	An important distal outflow could be a risk marker of progressive dilation and rupture.
					Pressure	
					Flow volume	
Karmonik* et al*. [43]	2012	1	CTA	CFD	WSS	High wall shear stress (>10 Pa) was observed for both assessments at the location of the entry tear. High stresses have the potential to cause additional injury to the endothelial cells, thereby potentially leading to tear progression and the creation of additional tears.
			MRI			
Chen* et al*. [9]	2013	1	CTA	FVM	WSS	The reduction of blood pressure in BMT patients lowers pressure and wall shear stress in the thoracic aorta in general, and flattens the pressure distribution on the outer wall of the dissection, potentially reducing the progressive enlargement of the false lumen.
			MRI			
Karmonik* et al*. [22]	2013	2	CTA	CFD	WSS	Maximum WSS was reduced at the site of largest dilation compared to healthy aorta.
			MRI			
Tolenaar* et al*. [44]	2013	60	CTA	None	Morphological data	The number of entry tears is a significant predictor for aortic growth. Patients with 1 entry tear at presentation show a higher growth rate than other patients.
Cheng* et al*. [47]	2013	4	CTA	FEM	TAWSS	There is a good correlation between high RRT regions and areas in the false lumen that subsequently thrombosed. RRT and turbulence intensity contours correlate well with subsequent areas of thrombus formation in the false lumen.
					RRT	
					OSI	
Dillon-Murphy* et al*. [49]	2016	1	CTA	FEM	WSS	The false lumen carries a greater proportion of descending aortic flow and is significantly larger than the true lumen. The false lumen exhibits a more homogenous pressure gradient along its length, with lower velocities and lower wall shear stress than the true lumen. Secondary communicating tears, particularly larger tears, have a significant impact on haemodynamics in the descending and thoracic aorta.
			MRI		TAWSS	
Ahmed* et al*. [45]	2016	14	Ideal *in vitro* model	FEM	Pressure	Larger distal tears decreased FL PP and FL MP, whereas smaller distal tears increased FL PP and FL MP. Larger proximal tears increased FL PP and FL MP, whereas smaller proximal tears decreased FL PP and FL MP.
					Flow states	
Long Ko* et al*. [50]	2017	1	CTA	FVM	WSS	High wall shear stress difference between true and false lumens infers the possible generation of descending aortic dissection along the aorta.
Chen* et al*. [9]	2013	1	CTA	CFD	Flow states	An obvious low wall shear stress zone was formed on false lumen wall near the entry tear, which was consistent with the thrombus position in the patient.
					Pressure	
					Shear stress	
Xu* et al*. [48]	2018	1	CTA	FEM	TAWSS	Low TAWSS is associated with deformation only below a threshold that may be correlated to biological dynamics in the arterial wall.
					RRT	
					OSI	
Bonfanti* et al*. [51]	2019	3	CTA ECHO	FVM	TAWSS	It was noted that small tears in the distal intimal flap induce disturbed flow in both lumina. Moreover, oscillatory pressures across the intimal flap were often observed in proximity to the tears in the abdominal region, which could indicate a risk of dynamic obstruction of the true lumen.
					OSI	
					RRT	

MRI, magnetic resonance imaging; OSI, oscillatory shear index; WSS, wall shear stress; CTA, computed tomography angiography; FVM, finite volume method; ECHO, echocardiography; RRT, relative residence time; FEM, finite element method; FL, false lumen; MP, inlet mean pressure; PP, pulse pressure; CFD, computational fluid dynamics; TAWSS, time-averaged wall shear stress; BMT, best medical treatment.

In addition to pressure and WSS, higher RRT values are present near the area of the celiac artery, which was positively correlated with local thrombosis, thereby contributing to true luminal blood flow obstruction and subsequent organ hypoperfusion [51]. Dillon-Murphy* et al*. [49] found that distal secondary tears were critical for reducing false lumen pressure and limiting dissection progression. This is consistent with the study by Tolenaar* et al*. [44] showing that patients with only a single or a smaller distal tear, exhibited unfavorable long-term hemodynamics, compared with those with multiple secondary tears [45, 49]. However, a larger distal tear may be a significant risk factor for progressive aortic expansion and rupture, as observed by CFD analysis conducted by Rudenick* et al*. [46] on 3 silicone models with different tear conditions. Additional factors affecting type B dissection progression are primary tear size and location, in which greater false luminal blood flow is associated with larger tears, as well as tears closer to the aortic arch. Increased false luminal enlargement has also been associated with larger TWSS around the tear, and contributes to further enlargement [47]. However, this association between larger TWSS and increased false luminal size was found by Xu* et al*. [48] to only be applicable for <2.5 dyn/${\mathrm{cm}}^{2}$. In addition, Osswald* et al*. [53] found that elevated WSS in the aortic wall adjacent to the left subclavian artery was a risk factor for retrograde type A aortic dissection (RTAD).

### 3.5 Application of Computational Fluid Dynamics for Evaluating Endovascular Techniques and Postoperative Outcomes

Endovascular surgeries, in the form of TEVAR or endovascular aneurysm repair (EVAR), have become an important treatment for type B aortic dissection and thoracic aortic aneurysms. However, TEVAR/EVAR has multiple complications, such as endoleaks, stent displacement/collapse, and RTAD. The occurrence of these complications had been related to the anatomical complexity of the dissection, aortic curvature, anchoring site conditions, and various other biomechanical indicators, all of which could be identified by CFD to aid in predicting their occurrence (Table 3, Ref. [7, 8, 9, 10, 23, 24, 54, 55, 56, 57, 58, 59, 60, 61, 62, 63, 64]). As a result, CFD technology could be a useful tool to evaluate the likelihood of complications from TEVAR/EVAR.

**Table 3.** **Characteristics of individual studies associated with operative outcomes**.

Authors	Year of publication	No. of cases	Imaging data	Modelling and simulation methods	CFD parameter	Key findings
Frauenfelder* et al*. [54]	2006	12	CTA	CFD	Flow Pattern	After stenting, the simulation shows a reduction of wall pressure and wall shear stress and a more equal flow through both external iliac arteries after stenting.
				FSI	WP	
					WSS	
Howell* et al*. [55]	2007	4	CTA	FVM	Pressure	Stent-grafts with short stiff limbs are probably less prone to proximal stent migration than stent-grafts with long floppy limbs. Oversizing may affect displacement force and migration risk.
Karmonik* et al*. [24]	2010	3	MRI	CFD	Pressure	EVAR treatment, by occluding the entrance tear may results in large pressure reduction in the false lumen effectively reducing complication risk.
					Flow Profiles	
Karmonik* et al*. [23]	2011	1	MRI	CFD	WSS	The maximum WSS was lowered post EVAR by more than a factor. Occlusion of the entrance tear by stent graft placement eliminated antegrade flow in the false lumen.
					dynP	
Karmonik* et al*. [56]	2011	1	MRI	CFD	Flow patterns	Chronic AD with outflow restrictions (partial FL thrombosis) may exhibit elevated FL pressures promoting lumen expansion and finally rupture, which is supported by clinical findings investigating the predictive power of partial FL thrombosis for survival.
					Pressure gradients	
Prasad* et al*. [62]	2011	1	CTA	FEM	Displacement forces	The predicted critical zone of intermodular stress concentration and frictional instability matched the location of the type III endoleak observed in the 4-year follow-up CT image.
				CSM	von Mises	
Shek* et al*. [8]	2012	1	CTA	CFD	WSS	Improved flow-related thrombosis resistance in the short term. There may be long-term fatigue implications to stent graft use in the cross configuration when compared to the direct configuration.
					TAWSS	
					OSI	
Chen* et al*. [9]	2013	1	CTA	FVM	Pressure	Reduction of blood pressure in BMT patients lowers pressure and wall shear stress in the thoracic aorta in general, and flattens the pressure distribution on the outer wall of the dissection, potentially reducing the progressive enlargement of the false lumen.
			MRI		WSS	
Pasta* et al*. [61]	2013	1	CTA	FEM	PE	Increased PE imparts an apparent risk of distal end-organ malperfusion and proximal hypertension and that both increased PE and θ lead to a markedly increased transmural pressure across the TASG wall, a load that would portend TASG collapse.
Alimohammadi* et al*. [57]	2014	1	CTA	CFD	WSS	Single stenting marginally decreased pressure and peak WSS values. Double-stent showed a 40% reduction in flow resistance, compared to just 1.5% for the single stent-graft.
				Windkessel	TAWSS	
					OSI	
Bogerijen* et al*. [60]	2014	1	CTA	FEM	Pressure	Protrusion extension conveys an apparent risk of distal end-organ malperfusion and proximal hypertension, being also proportional to a pressure load acting across the graft wall, potentially inducing stent-graft collapse.
					Flow patterns	
Xu* et al*. [59]	2017	2	CTA	FVM	WSS	False-to-true luminal pressure difference (PDiff) and particle relative residence time (RRT) are found related to FL remodeling.
					TAWSS	
					OSI	
					RRT	
Nauta* et al*. [64]	2017	1	CTA	FEM	PLAP	Regions of high PLAP were associated with aortic thrombus. Aortic repair resolved pathologic flow patterns, reducing PLAP. Branched endografting also relieved complex flow patterns reducing PLAP.
			MRI	Windkessel		
Costache *et al*. [7]	2018	1	CTA	CFD	Flow states	Multilayer Flow Modulator (MFM) implantation is a promising treatment for complicated TBAD due to the unique ability of these devices to stabilize the entire aortic wall without compromising the flow in the major aortic side branches.
Dottori* et al*. [63]	2020	8	CTA	CFD	Cross-section area	After EVAS technique, the pressure difference in the upper abdominal aorta of renal artery was large, and the flow velocity, WSS and reflux degree of iliac artery branch implant were higher than those of EVAR, which required close follow-up.
					Pressure	
					Blood velocity	
					WSS	
Mariscalco* et al*. [10]	2020	1	CTA	CFD	Flow states	Demonstrated a more physiological and stable cerebral blood perfusion when the carotid‐subclavian bypass is used as direct arterial inflow for cerebral perfusion.
					Blood velocity	
Li* et al*. [58]	2021	48	CTA	FEM	TAWSS	The different morphology of the re-entry tears had different effects on the thrombosis-related hemodynamic parameters in FL following TEVAR. The number of re-entry tears was most crucial to the potential thrombosis in the post-TEVAR FL of TBAD patients.
					OSI	
					ECAP	
					RRT	

CTA, computed tomography angiography; MRI, magnetic resonance imaging; FVM, finite volume method; OSI, oscillatory shear index; WSS, wall shear stress; RRT, relative residence time; FEM, finite element method; dynP, dynamic pressure; FSI, fluid-structure interaction; ECAP, endothelial cell activation potential; PE, protrusion extension, the angle between the TASG and the lesser curvature of the aorta; CSM, computational solid mechanics; PLAP, platelet activation potential; EVAR, endovascular aneurysm repair; CFD, computational fluid dynamics; WP, wall pressure; AD, aortic dissection; FL, false lumen; CT, computed tomography; TAWSS, time-averaged wall shear stress; BMT, best medical treatment; TASG, thoracic aortic stent graft; TBAD, type B aortic dissection; TEVAR, thoracic endovascular aortic repair; EVAS, endovascular aneurysm sealing.

One example regarding the application of CFD was performed in 2006, when Frauenfelder* et al*. [54] established a silicone model, based on CTA data from 11 aortic aneurysm patients, to develop CFD simulation using the fluid-structure interaction method (FSI). The quantitative results indicated that the blood flow in the descending aorta and the iliac arteries were more uniform, the flow field was smoother, and the size and number of eddy currents were reduced after stent implantation [54]. In addition to the characterization of the general flow field after stent implantation, other studies have suggested that short, rigid, and strong branch stents were able to transmit forces from the stent bifurcation to the aortic bifurcation, which was impossible with long, flexible stents. Therefore, all displacement forces at the bifurcation must be borne by the stent trunk and the infrarenal aneurysm neck anchoring this area, leading to higher wall stress and shear stress around the stent bifurcation area, which may serve as the mechanical basis behind long-term stent displacement and endoleaks [54].

Karmonik* et al*. [24], using MRI-based CFD to simulate proximal and distal tear closure for type B dissection, found that TEVAR was able to effectively reduce false luminal antegrade blood flow. The total false lumen pressure decreased significantly (by approximately 97%), and the maximum WSS in the false lumen was reduced, all of which could lower the incidence of long-term complications. However, it was found that a short-term reversal of the pressure difference between the true and false lumens occurred at the end of systole, in which the pressure in the false lumen was higher than that in the true lumen. This may serve as the basis for some type B dissections, with weak flaps, being associated with the long-term risks of stent rupture, displacement, and endoleaks [23, 24, 55]. In contrast, distal tear closure or thrombosis could result in increased false lumen pressure, thus increasing the long-term risk of rupture [45, 56]. Chen* et al*. [9] found in a follow-up simulation study, conducted on a type B dissection patient, that separate closures of either the proximal or distal tear were unable to reduce false luminal perfusion, and that stent coverage was required. The requirement for stent coverage was further supported by Alimohammadi* et al*. [57], who found that compared to having a single stent covering the proximal rupture, double stents covering both proximal tears reduced descending aorta flow resistance by 40%, and significantly attenuated elevated WSS in that area. The number of distal tears was determined to be a key factor for occurrence of false lumen thrombosis. Therefore, to promote thrombosis, it is recommended to selectively perform a one-stage repair of distal tears with large areas, and those located in non-visceral arterial branches [58].

Xu* et al*. [59] conducted a study on the risk factors for further FL expansion after TEVAR. They found that the stable and progressive patients after TEVAR had significant differences in WSS, RRT, and true and false lumen pressure. A significant increase in the true-false lumen pressure difference suggests a potential expansion of the FL. Therefore, early monitoring of the pressure differences, identifying the false lumen entrance location, and measuring maximal pressure difference could aid in predicting the onset of false luminal expansion [59]. The positioning of the TEVAR stent could also affect the occurrence of endoleaks, in which it is often anchored at the proximal end of the aortic arch, when the tear position is high and close to the orifice of the left subclavian artery. This results in the left subclavian artery blood supply being sacrificed, though it did not significantly increase abnormal aortic arch blood flow, as documented by Van Bogerijen* et al*. [60]. However, this “bird-beak” change increased the risk of poor distal organ perfusion, proximal aortic hypertension, long-term stent collapse, and type I endoleaks [60, 61]. These findings were supported by a follow-up study by Prasad* et al*. [62] on a patient who developed long-term type III endoleaks after receiving two TEVAR stents. It was observed that the highest stress was located at the junction between the two stents. Furthermore, tribological stability testing showed that most of the surface area (53% of this region) had unstable contacts, corresponding to the location of the Type III endoleaks [62].

The cross-limb EVAR stent has higher helical blood flow and is able to reduce stent thrombosis. However, EVAR stents are associated with higher stress fluctuations, which may cause stent fatigue and subsequent long-term device failure [8]. Due to these limitations for EVAR, open surgery may be considered for abdominal aortic aneurysms with short necks and lesions close to the renal artery orifice. Open surgery was found by CFD simulations to significantly reduce abdominal aortic false luminal retrograde blood flow, compared to EVAR. However, iliac arterial retrograde blood flow, flow rate, and WSS were all significantly increased [63]. Additionally, after replacing the ascending aorta and total arch replacement with the frozen elephant trunk technique, distortion of the graft is the main factor affecting ascending aorta hemodynamics and thrombosis. Studies have shown that the platelet activation potential index could be significantly reduced if the artificial vessel maintained its proper shape and surface smoothness [64].

### 3.6 Application of Computational Fluid Dynamics Technology in Type A Aortic Dissection

Independent risk factors for predicting early mortality from type A aortic dissections include age, previous cardiac surgery, hypotension/shock, cardiac tamponade, pulselessness and myocardial ischemia/infarction. CFD has proven to be useful for obtaining hemodynamic indices, which have provided new directions for developing prediction models of adverse events associated with type A dissections (Table 4, Ref. [3, 65, 66, 67]). Table 4 summarizes the characteristics of individual studies associated with type A aortic dissection (TAAD).

**Table 4.** **Characteristics of individual studies associated with TAAD**.

Authors	Year of publication	No. of cases	Imaging data	modelling and simulation methods	CFD parameter	Key findings
Malvindi* et al*. [3]	2017	1	CTA	CFD	WSS	An abnormal helical flow pattern inside the aneurysm and an increased wall stress on the right postero-lateral wall of the ascending aorta. These values were largely higher than the theoretical cut-off for aortic wall dissection and confirmed during the operation for dissection repair.
				FSI		
Chi* et al*. [65]	2017	7	CTA	CFD	WSS	Dilation of the ascending aorta and alterations in the branching angles may be the key determinants of a high WSS that leads to type A dissection. Greater tortuosity of the aortic arch leads to stronger helical flow through the distal aortic arch, which may be related to tears in this region.
Xiao* et al*. [67]	2018	20	CTA	CFD	WSS	The blood flow velocity and aortic branch vessels faster, the rate of organ mal-perfusion is lower. The aorta and branch vascular wall shear stress increases, the rate of adverse postoperative organ perfusion is lower.
Ma* et al*. [66]	2021	20	CTA	FEM	MWP	The uneven distribution of WSS and VS play an important role in the rupture of AD. Eddy viscosity (EV) demonstrates powerful predictive value in the rupture of aortic dissection.
					MWSS	
					MVS	
					MEV	
					MAWP	
					MAVS	

CTA, computed tomography angiography; FEM, finite element method; WSS, wall shear stress; FSI, fluid-structure interaction; MWP, mean wall pressure; MWSS, mean wall shear stress; MVS, mean vortex strength; MEV, mean eddy viscosity; MAWP, maximum wall pressure; MAVS maximum vortex strength; TAAD, type A aortic dissection; CFD, computational fluid dynamics; VS, vortex strength; AD, aortic dissection.

Due to type A aortic dissections being associated with profound pathological changes and complex morphological variations, particularly with respect to multiple irregular ruptures, as well as the involvement of the aortic sinus and branch vessels above the arch, it has been extremely difficult to construct a 3D model suitable for CFD simulation. This is further complicated by the fact that most type A dissection patients cannot undergo MRI examination due to the acuteness and instability of their conditions. As a result, the application of CFD approaches for this disease has been hampered, owing to complicating factors, such as the large number of branch outlets and errors in the setting of the outlet boundary, adding another layer of complexity to the development of a CFD model.

Nevertheless, systematic hemodynamic analyses of type A aortic dissection, using CFD techniques, have been conducted. Malvindi* et al*. [3] used a simple CFD technique as part of their follow-up of a patient who initially presented with an ascending aortic aneurysm and later developed type A dissection to perform transient peak simulations of pre- and post-dissection conditions. The results of these simulations showed that abnormal spiral flow was present in the ascending aortic aneurysm, along with significant increases in wall stress and WSS for the right posterior portion of the ascending aorta, both of which corresponded with the site of the dissection flap. This suggests that regions with high wall stress and WSS are the most prone to developing intimal damage and tears in type A aortic dissections [3].

In another analysis, Chi* et al*. [65] used CFD for type A aortic dissections in five patients, in which the dissecting flap was artificially removed to simulate pre-dissection conditions. They found that increases in ascending aortic diameter corresponded to increased mean WSS, and the area was related to the actual tear site [65]. Furthermore, the progression of the ascending aortic diameter was still a risk factor for predicting long-term dissection in aortic aneurysm patients. These findings were further supported by Ma* et al*. [66], who found that the mean wall pressure, mean WSS, mean vortex strength, and mean eddy viscosity of patients who died from type A dissection were significantly higher than those who survived. Mean eddy viscosity was found under multivariate logistic regression analysis to be an independent predictor of in-hospital mortality (*p* = 0.037, AUC = 0.94) [66]. CFD simulation was used by Xiao* et al*. [67] to study the problem of poor liver and kidney perfusion in post-type A aortic dissections, and showed that increased branch vessel flow velocity and higher branch vessel wall WSS were associated with lower probabilities of poor organ perfusion post-surgery.

Even though few studies exist for type A aortic dissection CFD hemodynamics, these studies have proven the feasibility of CFD for studying the type A aortic dissection, which could reflect blood flow field characteristics, both pre- and post-dissection, as well as providing quantitative calculations for biomechanical indicators, which can provide assistance in developing risk prediction approaches for type A aortic dissections in the future.

### 3.7 Current Limitations of Computational Fluid Dynamics

CFD is a very promising analysis method. It can transform the complexity of human vessels and blood flow into a simplification of mathematical models, giving computational fluid dynamics the potential to enhance standard medical images and thus aid in treatment decisions. However, at the present stage, it requires a lot of labor and computing time, making it difficult to calculate the results in a short time and immediately put into a wide range of clinical application. Another major disadvantage is that the biomechanical data obtained can only be used for intra-group comparison because the early boundary conditions were set differently across teams. In the future, with the further improvement and consensus of the algorithm and branch vessel outlet pressure, rate and resistance, unified calculation can be completed for mutual comparison and evaluation.

## 4. Conclusions

CFD has been found to be a feasible and accurate simulation method for evaluating the biomechanical characteristics of aortic diseases and surgeries, which can be used for clinical diagnosis, treatment, scientific research, and device development. Over the past two decades, we have gained a comprehensive and in-depth understanding of the hemodynamics and biomechanics affecting aortic disease. CFD, based on phase-contrast magnetic resonance imaging (PC-MRI) and CTA, has emerged as a valuable non-invasive assessment technique able to assess and visualize the intricate details of aortic blood flow patterns, both qualitatively and quantitatively.

## Supplementary Material

Supplementary material associated with this article can be found, in the online version, at https://doi.org/10.31083/j.rcm2412355.
